# Effects of different stent wire mesh densities on hemodynamics in aneurysms of different sizes

**DOI:** 10.1371/journal.pone.0269675

**Published:** 2022-06-10

**Authors:** Shunsuke Masuda, Soichiro Fujimura, Hiroyuki Takao, Kohei Takeshita, Takashi Suzuki, Yuya Uchiyama, Kostadin Karagiozov, Toshihiro Ishibashi, Koji Fukudome, Makoto Yamamoto, Yuichi Murayama

**Affiliations:** 1 Department of Innovation for Medical Information Technology, The Jikei University School of Medicine, Tokyo, Japan; 2 Cybernet Systems Co., Ltd., Tokyo, Japan; 3 Department of Mechanical Engineering, Tokyo University of Science, Tokyo, Japan; 4 Department of Neurosurgery, The Jikei University School of Medicine, Tokyo, Japan; 5 Graduate School of Mechanical Engineering, Tokyo University of Science, Tokyo, Japan; 6 Digital Health & SYNGO Department, Siemens Healthcare K.K., Tokyo, Japan; RKH Klinikum Ludwigsburg, GERMANY

## Abstract

**Background:**

Intracranial stents are used to treat aneurysms by diverting the blood flow from entering into the aneurysmal dome. Although delayed rupture is rare, clinical outcomes are extremely poor in such cases. Hemodynamics after stent deployment may be related to delayed rupture and a better understanding of the basic characteristics of pressure changes resulting from stent deployment is needed; therefore, this study investigated the relationships between hemodynamics in aneurysms of different sizes treated using stents of different wire mesh densities.

**Methods:**

Using computational fluid dynamics analysis, parameters related to velocity, volume flow rate, pressure, and residual volume inside the aneurysm were evaluated in digital models of 5 basic aneurysms of differing sizes (Small, Medium, Medium-Large, Large, and Giant) and using 6 different types of stent (varying number of wires, stent pitch and wire mesh density) for each aneurysm.

**Results:**

Regardless of the aneurysm size, the velocity inside the aneurysm and the volume flow rate into the aneurysm were observed to continuously decrease up to 89.2% and 78.1%, respectively, with increasing stent mesh density. In terms of pressure, for giant aneurysms, the pressure on the aneurysmal surface elevated to 10.3%, then decreased to 5.1% with increasing stent mesh density. However, in smaller aneurysms, this pressure continuously decreased with increasing stent mesh density. The flow-diverting effect of the stents was limited when a stent with low mesh density (under 20%) was used with a giant aneurysm.

**Conclusions:**

The present results indicate that the selection of appropriate stents according to aneurysm size may contribute to reduced risks of hemodynamic alternations related to stent deployment, which could reduce the incidence of delayed rupture.

## Introduction

Flow diverter (FD) stents are designed to divert blood flow from entering into the aneurysmal dome with their high wire mesh density. Although FDs offer an effective device to treat large, wide-necked, dissecting, and fusiform aneurysms, postoperative complications such as parent artery occlusion, in-stent stenosis, thromboembolism, branch artery occlusion, and delayed rupture have been reported [1–9]. In particular, clinical outcomes are extremely poor when delayed rupture occurs, even though the frequency of this event is under 1.1% [9–14].

Computational fluid dynamics (CFD) has been widely applied to research the hemodynamics in cerebral arteries. In particular, results from CFD research have suggested that hemodynamics after stent deployment may be related to delayed rupture. Although these hemodynamic changes in post-stent deployment can also be associated with the aneurysm location and shape, some researchers have focused on the changed flow due to the deployed stent. Cebral et al. performed CFD of pre- and post-treatment states for 7 aneurysms, including 3 giant aneurysms that ruptured after stent deployment and 4 successfully treated aneurysm [15]. Their results indicated that FD deployment can cause pressure elevation within the aneurysm, potentially contributing to rupture. Meanwhile, Larrabide et al. studied 23 patients for conditions before and after stent deployment using CFD [16]. As a result, no significant aneurysmal mean or peak pressure changes were observed, while other hemodynamic variables including wall shear stress and velocity exhibited more significant changes. Stent deployment has also been reported to reduce pressure inside the aneurysm [17]. The effects of such increases in pressure due to stent deployment remain controversial. On the other hand, pressure effects from hemodynamics may provide a greater contribution to conditions at the aneurysmal wall. Suzuki et al. analyzed hemodynamics in 50 unruptured middle cerebral artery aneurysms treated by clipping [18]. They revealed that spatial and temporal maximum pressure areas corresponded with regions of thin aneurysmal walls in 41 cases. Due to the potential risks of pressure elevations, a better understanding of the basic characteristics of pressure changes resulting from stent deployment is needed, to ensure efficient and safe interventions. In addition, the concept that investigation of basic hemodynamic changes using CFD may facilitate optimal stent design to treat aneurysms with higher success rates is very important.

In the present study, we investigated the relationships between hemodynamics in aneurysms of different sizes treated using stents of different wire mesh densities. CFD analysis was performed on 5 basic aneurysm models differing in size, with and without stents of 6 different wire mesh densities. We then evaluated hemodynamic parameters including pressure, velocity, volume flow rate, and residual volume in the aneurysm.

## Materials and methods

### Basic aneurysmal models

**Fig 1:**
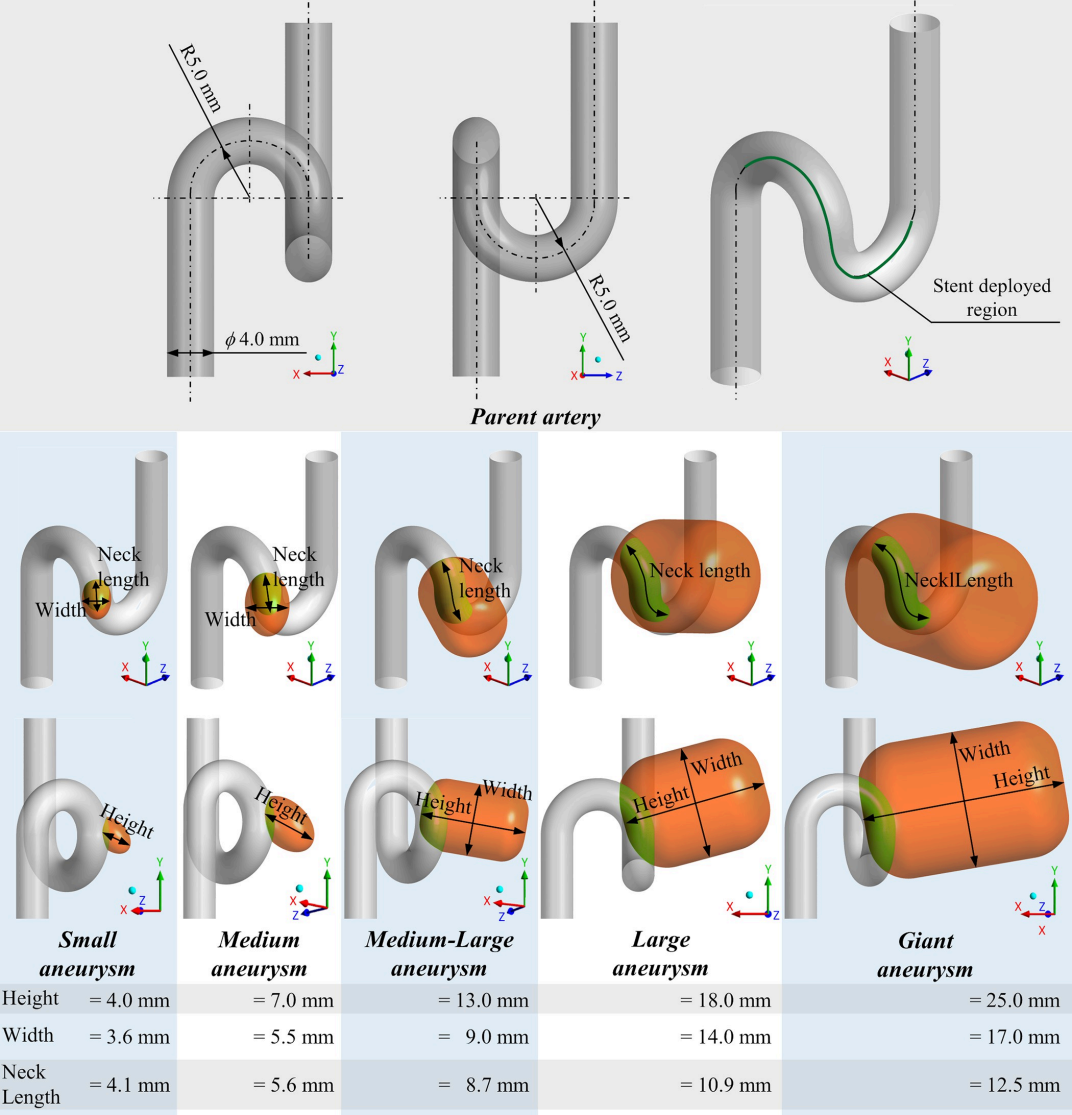
Generated basic aneurysm models and parent artery. The stent was deployed in the region of the green centerline. Five types of aneurysms (Small, Medium, Medium-Large, Large, and Giant) were generated by varying aneurysmal height, width, and neck length.

We designed 5 basic aneurysm models of different sizes to imitate cavernous aneurysms of the internal carotid artery with varying aneurysmal height, width, and neck length (i.e., Small, Medium, Medium-Large, Large, and Giant). Based on several previous studies, we classified the height of aneurysms into five categories: 4.0 mm, 7.0 mm, 13.0 mm, 18.0 mm, and 25.0 mm [19–21]. The cylindrical shape was adopted because in actual clinical settings, the shape of cerebral aneurysms is more oval than spherical, as reported by an average aspect ratio (AR) value of 1.1–1.6 [22]. The parent artery with a diameter of 4 mm was bent twice at 180° each turn, imitating a “siphon” of the internal carotid artery. These models were generated using DesignModeler on ANSYS Workbench 2020R1 software (ANSYS Inc., Canonsburg, PA, USA). Details of the parent artery and aneurysm model are shown in Fig 1.

### Deployed stent designs

**Fig 2:**
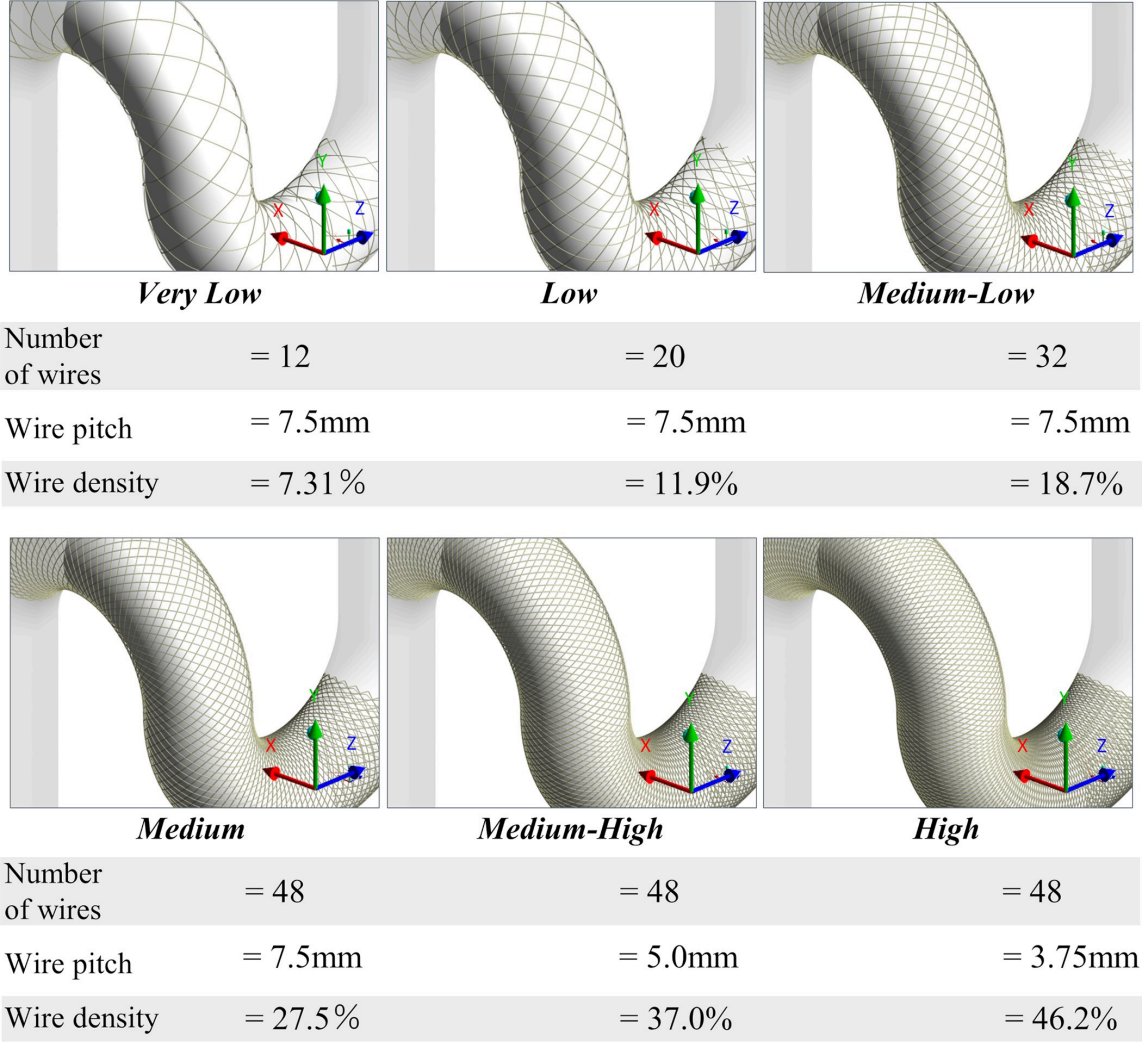
Generated 6 types of deployed stents. The Stents were generated by varying the number of wires and wire pitch, and the wire density was calculated.

Various stent models were generated with varying numbers of wires. With reference to the Pipeline Embolization Device (PED) (Covidien/Medtronic, Irvine, CA, USA), which has 48 wires braided in a mesh and shows 42.1% wire mesh density in our basic model, we fully modeled 6 types of stents using ANSYS Mechanical 2020R1 (ANSYS Inc.) as shown in Fig 2; characteristics of the Very Low, Low, Medium-Low, Medium, Medium-High, and High stent types are shown in Table 1. As the artery was curved, a toroidal coordinate system was used to generate the stent structure by generating “wrapping” of the stent wire around the surface of the artery without any structure simulation (the geometry was created using only geometrical methods). Since the toroidal coordinate system represents positions on the surface of a donut shape, it is possible to generate the stent structure by specifying the stent pitch and number of wires. Therefore, the generated stent reflects the change in wire density on the inner and outer surfaces of the curve of the stent (i.e., the wire density is relatively higher on the concave and lower on the convex surface). The diameter of the stent wire was 0.03 mm in all stents, the same as in PED. Modeled stents were deployed at the same position of the parent artery (Fig 1) and the aneurysmal neck was fully covered by the stent.

**Table 1:** Number of wires, stent pitch, and wire mesh density for each modeled stent type.

Stent type	Number of wires	Stent pitch [mm]	Wire mesh density [%]
Very Low	12	7.5	7.31
Low	20	7.5	11.9
Medium-Low	32	7.5	18.7
Medium	48	7.5	27.5
Medium-High	48	5.0	37.0
High	48	3.75	46.2

### CFD analysis

We generated a computational unstructured grid using ANSYS ICEM CFD 2020R1 (ANSYS Inc.) based on geometric data of the basic aneurysm models and deployed stents. The generated mesh comprised tetra mesh (maximum size 0.3 mm) and 7 layers of prism mesh in the vicinity of vessel walls (the first layer having a thickness of 0.02 mm and the remaining seven layers having a total thickness of 0.3 mm). A grid independence study was performed on our previous study and confirmed that the grid size was adequate to obtain the comparison results independent of the grid size [23]. The total number of elements in the meshes ranged from 4.1 to 7.6 million. The flow field was assumed to comprise incompressible laminar flow and blood was assumed to act as a Newtonian fluid with density of 1,100 kg/m^3^ and dynamic viscosity of 0.0036 Pa·s. Blood flow analysis was performed using ANSYS CFX 2020R1 (ANSYS Inc.). For basic aneurysmal models with and without stents, unsteady flow analysis was performed by imposing the spatial averaged volume flow rate at the inlet, as measured at the internal carotid artery in healthy adults by Ford et al. with a time step of 5×10^−4^ s, and results were output every 0.05 s [24]. At the outlet, relative static pressure was fixed at 0 Pa. 75 mm extended tubes were connected to all inlets and outlets to obtain fully developed inflow and avoid the effect of outlet boundary. Since the peak Reynolds number at the parent artery did not exceed 800, a turbulence model was not applied. When we performed blood flow analysis in stent-deployed models, the overset grid method was applied to define the stent as a rigid wire. The aneurysm and parent artery wall were also assumed to act as a single rigid body. These boundary conditions have been validated and used in previous studies [25].

### Evaluation parameters

Some evaluation parameters were defined to evaluate the relationship between aneurysmal size and stent wire mesh density. As basic parameters to identify hemodynamics in the aneurysm, we measured spatial average velocity in the aneurysmal dome, volume flow rate into the aneurysm through the aneurysmal neck, and pressure on the aneurysm dome wall. All these parameters were sampled from the second pulsation and temporally normalized through the second pulsation time. Relative changes of these parameters were observed to identify hemodynamic changes between pre- and post-stent deployment, defined as relative change of velocity (*RC-V*), volume flow rate (*RC-VFR*), and pressure (*RC-P*) as shown in the following equations:

$$RC‐V=\frac{{averaged\;velocity\;in\;aneurysm}_{post}}{{averaged\;velocity\;in\;aneurysm}_{pre}}-1$$ (1)

$$RC‐VFR=\frac{{volume\;flow\;rate\;into\;aneurysm}_{post}}{{volume\;flow\;rate\;into\;aneurysm}_{pre}}-1$$ (2)

$$RC‐P=\frac{{averaged\;pressure\;at\;aneurysm}_{post}}{{averaged\;pressure\;at\;aneurysm}_{pre}}-1$$ (3)

where the subscript notation *pre* indicates the state before stenting and *post* indicates the state after stenting. In addition, to evaluate flow stagnation following stent deployment, residual blood volume in the aneurysm was also evaluated as residual volume ratio (*RVR*).

$$RVR=\frac{residual\;flow\;volume}{aneurysm\;volume}$$ (4)

where *residual flow volume* was calculated by subtracting the replacement flow rate from the aneurysm volume after computing two cardiac cycles.

## Results

### RC-V and RC-VFR show continuous decreases

**Fig 3:**
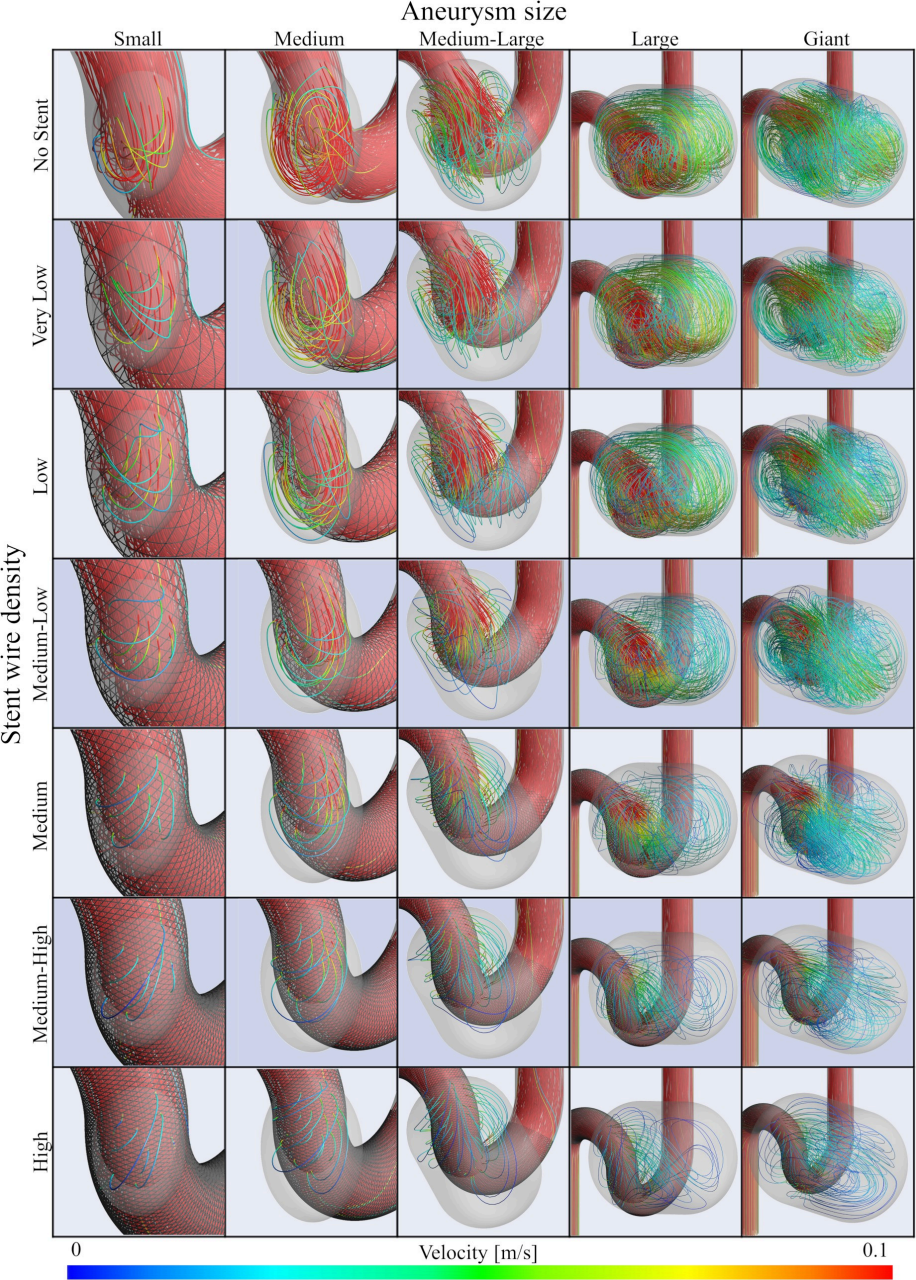
Streamlines before and after stent deployment in each aneurysm size and stent wire mesh density. The streamlines are sampled from the second systole and colored in the range of 0–0.1 m/s according to the velocity.

**Fig 4:**
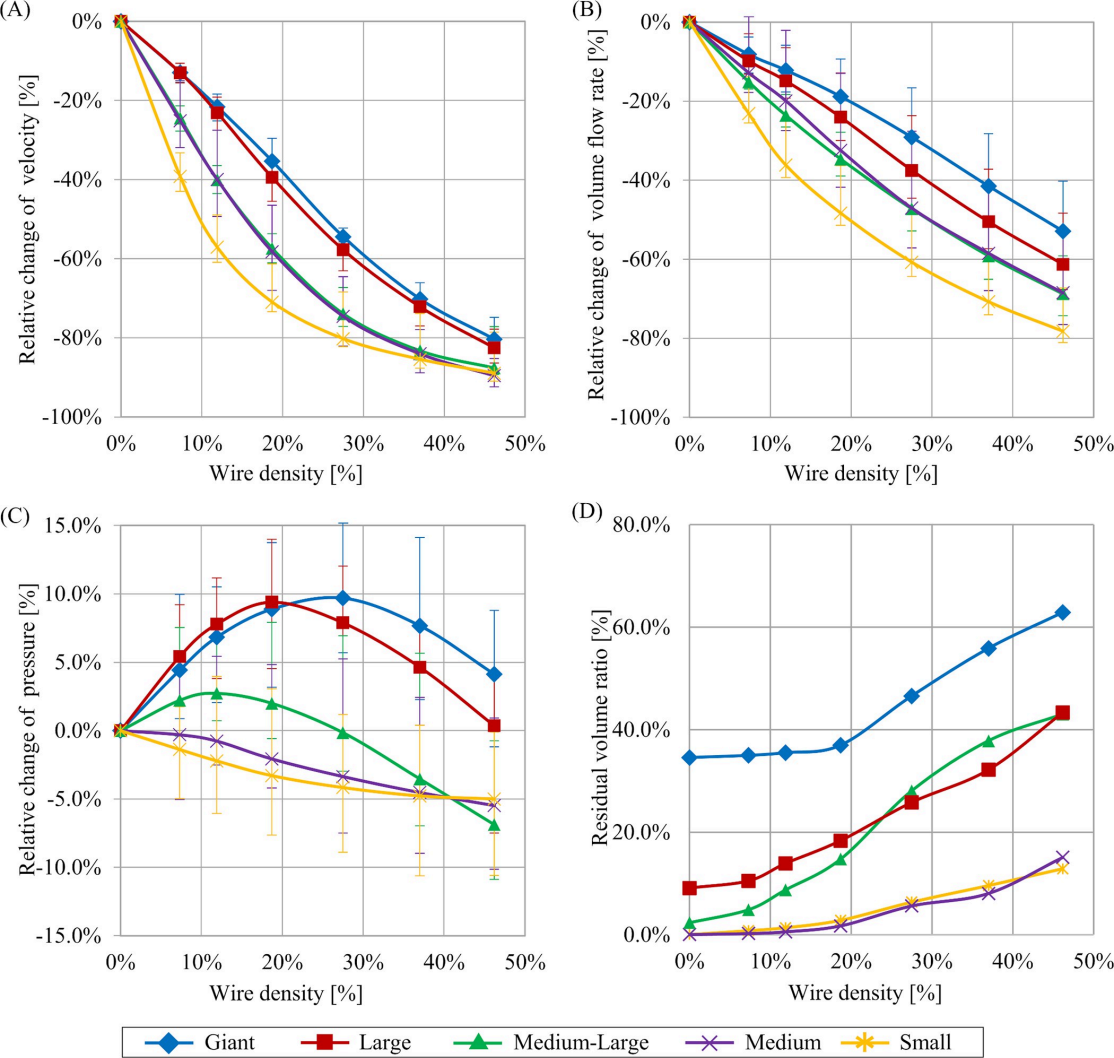
Relationships between each parameter relative change and stent wire mesh density. The range of relative change over the second pulsation observed in unsteady flow simulation are shown in whiskers. A) Results of relative change of velocity (*RC-V*). B) Results of relative change of volume flow rate (*RC-VFR*). C) Results of relative change of pressure (*RC-P*). D) Results of residual volume ratio (*RVR*).

Streamlines for each combination of aneurysm size and stent variation, as sampled from the second systole in the analyzed pulsation, are summarized in Fig 3. We qualitatively observed that flow into the aneurysm decreased more when stents of higher wire mesh density were deployed. In addition, blood flow more easily entered the aneurysmal dome as aneurysm size increased. To quantitatively evaluate velocity and volume flow rate, results for *RC-V* and *RC-VFR* were measured, as summarized in Fig 4A and 4B. Decreased blood flow into aneurysms was also apparent from the graph (i.e., velocity in the aneurysm and volume flow rate into the aneurysm decreased continuously with increases in the wire mesh density of deployed stents). Maximum *RC-V* was -89.6% when the stent of highest wire mesh density (46.2%) was deployed in the Medium aneurysm. Similarly, maximum *RC-VFR* (-78.2%) was observed when the stent with 46.2% wire mesh density was deployed in the Small aneurysm. Although *RC-V* was indicated to be around -85% with the stent of highest wire mesh density regardless of aneurysm size, characteristics of the line of wire mesh density from 0% to 46.2% differed depending on aneurysm size. For example, in Small aneurysms, the yellow line in Fig 4A follows a downward-oriented convex parabola, and *RC-V* decreased sharply to -71.0% until wire mesh density reached 18.7%. However, *RC-V* then gradually changed to -89.0% until wire mesh density reached 46.2%. On the other hand, in the case of Giant or Large aneurysms, *RC-V* showed a relatively linear decrease from 0% to 80.4% or 82.5%, respectively, with increasing wire mesh density. The *RC-V*s of Medium-Large and Medium aneurysms were located between those of Large, Giant and Small aneurysms (Fig 4A).

### Pressure on the aneurysm wall showing non-continuous change

**Fig 5:**
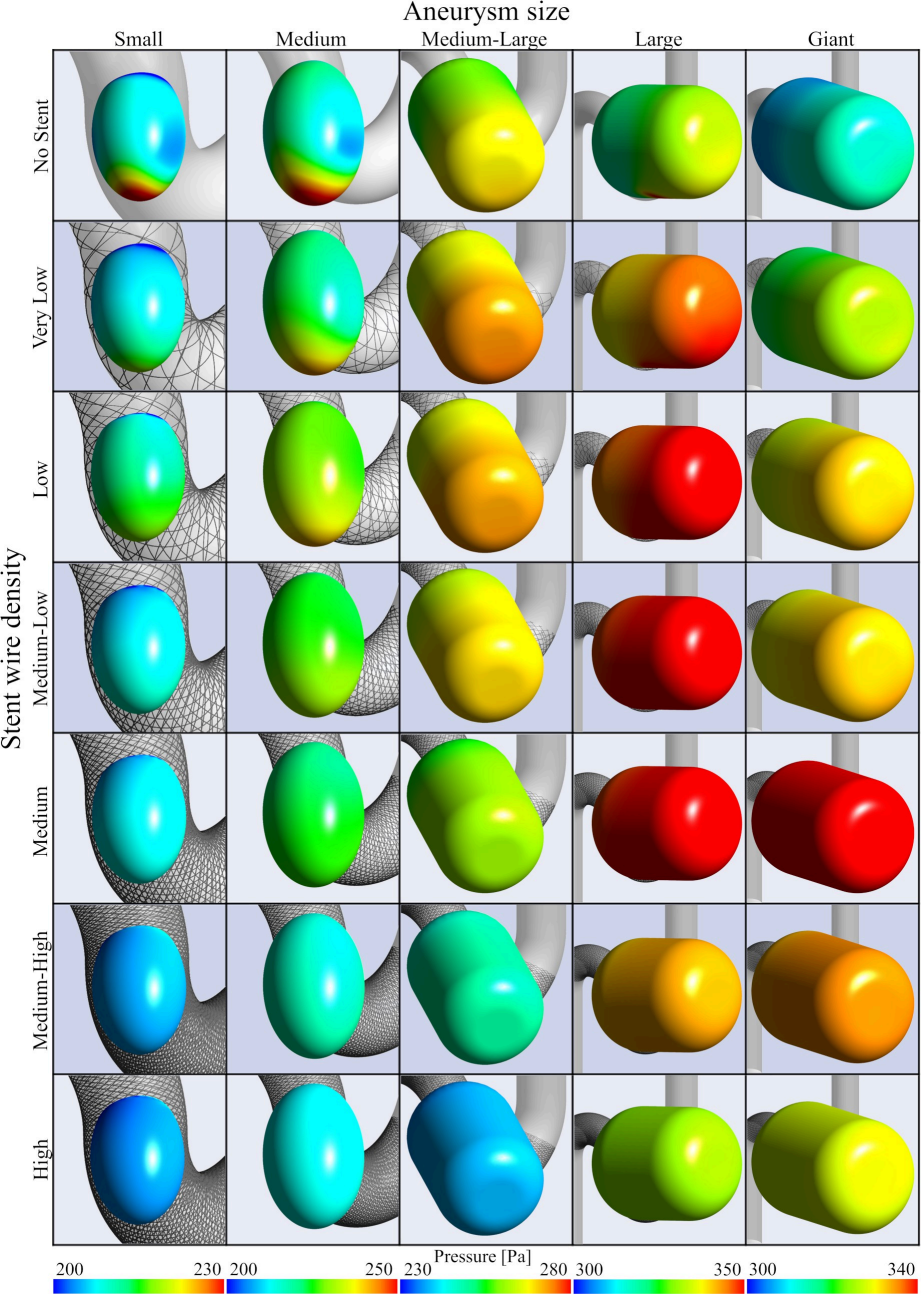
Pressure on aneurysmal surface before and after stent deployment for each aneurysm size and stent wire mesh density. The wall of aneurysms is sampled from the second systole and colored according to the pressure.

Pressure on the aneurysm wall was also sampled from second systole in the analyzed pulsations, as summarized in Fig 5. It should be noted that pressure values are relative values in each of the aneurysm cases. Pressure on the aneurysm wall was not constantly distributed in cases of No Stent or stents with Low wire mesh density. In particular, a high-pressure region was identified around the apex of the aneurysm where flow collision had occurred. The results of quantitative evaluation are summarized as *RC-P* in Fig 4C. *RC-P* decreased continuously in cases of Small and Medium aneurysms in the same manner as *RC-V* and *RC-VFR*, but tended to drop after elevation for aneurysms of Medium-Large size and above. More specifically, *RC-P* in a Giant aneurysm followed an upwardly turned convex parabola, and *RC-P* was elevated to 9.7% until wire mesh density reached 27.5%, then decreased to 4.1% until wire mesh density reached 46.2%. Although the same trends were observed in Large and Medium-Large aneurysms, *RC-P* finally decreased to 0.4% and -6.9%, respectively.

### Residual blood volume in aneurysms after two pulsations

The quantitative results of *RVR* are summarized in Fig 4D. Since smaller aneurysm show higher blood turnover, the *RVR* of Small and Medium aneurysms was around 15% for both after the two pulsations, even for a wire mesh density of 46.2%. Considering the slowing of blood volume turnover as aneurysm size increased, the *RVR* in Giant aneurysm was 34.5% even when a stent was not deployed. Relationships between *RVR* and wire mesh density showed different behaviors depending on aneurysm size. In Small, Medium, Medium-Large, and Large aneurysms, *RVR* showed continuous increases with increasing wire mesh density. On the other hand, in the case of Giant aneurysms, *RVR* changed by only 2.4% for wire mesh density within a range of 0–18.7%, then increasing further to 62.9% until wire mesh density reached 46.2%.

## Discussion

### Velocity and volume flow rate reduction due to stent deployment

Intracranial stents have been applied alone or to assist coil embolization in aneurysm treatment. In particular, FDs are now becoming one of the most popular devices, since they excel at diverting and shutting off blood flow with their higher wire mesh density compared to other intracranial stents. Since surgeons need to safely achieve effective endovascular treatment, they require reliable methods for selecting the optimal stent among the broad variety available for each specific aneurysm [26–29]. However, the hemodynamic effects of stent deployment remain poorly understood. In general, blood inflow into the aneurysm is believed to be more effectively blocked when a stent with higher wire mesh density is deployed, which often results in satisfactory occlusion of the aneurysms [30–32]. Our results also demonstrated that blood velocity in the aneurysm and volume flow rate into the aneurysm continuously decreased with increasing wire mesh density of the stent, regardless of aneurysm size. On the other hand, since the characteristics of velocity reduction differed depending on aneurysm size, a stent of somewhat lower wire mesh density may be sufficient to obtain the same reduction in velocity. Our results imply that even a stent of low wire mesh density can provide efficient reductions in velocity for smaller aneurysms. This perspective may provide a valuable guide to selecting the stent offering the necessary and sufficient velocity reduction effect, since high wire mesh density, including in FD stents, has also been reported as a risk factor for complications such as arterial branch occlusion. Rangel-Castilla et al. reported that the occlusion rate of arterial branches covered with an FD stent was 10.5% at the ophthalmic artery, and 10.7% at the posterior communicating artery [7]. Brinjikji et al. also indicated a perforator infarction rate of 3% and an ischemic stroke rate of 6% after FD stent treatment, according to a meta-analysis of 1654 aneurysms in 29 studies [8].

### Relationships among pressure change, aneurysm size, and stent wire mesh density

Unlike the graphs for velocity and volume flow rate, except for the cases of Small and Medium aneurysms, pressure changes did not show a continuous decrease. Instead, pressure elevated and then decreased with increasing wire mesh density. The following four possible reasons were considered for the hemodynamic loss that occurred for flow when entering an aneurysm with a deployed FD stent: A) loss generated during passage through the stent mesh from parent artery to aneurysm sac; B) loss generated during passage through the aneurysm sac; C) loss generated during passage through the stent mesh from aneurysm sac to parent artery; or D) confluent loss generated during the return to main blood flow in the parent artery. These losses occur on the upstream side in the sequence of (A), (B), (C), and (D). Intra-aneurysmal pressure will therefore rise if losses on the downstream side are relatively larger than the loss of A (i.e., pressure easily elevates when the total loss from aneurysm to parent artery is large, which indicates that inflow is easier than outflow). Conversely, pressure decreases when losses on the downstream side are relatively small, because inflow is difficult and outflow is easy. In other words, the balance of hemodynamic loss between inflow and outflow decides the trend in pressure changes. In the present study, intra-aneurysmal pressure elevation was observed in aneurysms of Medium-Large or above. When the aneurysm is larger, inflow is easier and inflow rate thus also become larger (Fig 3). Simultaneously, the total loss after passing through the stent from the parent artery can also be considered to enlarge, since aneurysm volume. Deploying a stent of low wire mesh density under such circumstances creates the possibility for intra-aneurysmal pressure elevation since the total loss after passing through a stent mesh from parent artery to aneurysm sac tends to be larger, although blood flow easily enters the aneurysm due to small loss of (A). However, as wire mesh density increases, aneurysm pressure drops after reaching a maximum, since the loss of (A) becomes higher. On the other hand, smaller aneurysm inflow is difficult and volume inflow rate is lower compared to larger aneurysms. Thus, even when the loss of (A) is small in a stent with a low wire mesh density, pressure can continue to decrease as inflow reduces.

### Blood flow stagnation after deployment of stents of different wire mesh densities in aneurysms of various sizes

One of the main concepts in stent deployment is to divert blood flow by the stent wire mesh. In particular, FD stents are intended to cause stagnant flow in aneurysms by using a high wire mesh density to induce thrombosis within the aneurysm. Our hemodynamic results indicate that the flow-diverting effects of stent deployment are limited in some cases, depending on aneurysm size and wire mesh density. Specifically, the Giant aneurysm model showed that *RVR*, indicating flow stagnation after stent deployment, did not increase significantly until wire mesh density reached around 20% (see Fig 4D) although the effect on velocity and volume flow rate into the aneurysm was confirmed as shown in Fig 4A and 4B. As a reference, the wire mesh density of PED calculated for our aneurysm model showed a value of 42.1%. It is to be noted that stents with low wire mesh density, such as those used in stent-assisted coiling, may have almost no significance in terms of stagnation effect, especially for large aneurysms. In addition, the same phenomenon of insufficient flow stagnation may occur in actual clinical settings using FD stents of high wire mesh density, depending on the specific geometry of the parent artery or aneurysm, since the wire mesh density can be locally reduced (e.g., the wire mesh density on the outer side of the stent curvature is lower in a deployment in a curved artery).

### Novelty and importance of this study based on pressure changes and blood flow stagnation comparing to previous studies

Although this study includes some limitations that will be discussed later, we believe that it provides a new interpretation of intra-aneurysmal pressure and blood flow stagnation after stent deployment, considering what has been already reported until now. Cebral et al. reported on results of CFD analysis, finding that pressure elevates within the aneurysms as a result of FD deployment [15]. Conversely, Larrabide et al. indicated that no significant aneurysmal mean or peak pressure change was observed while other hemodynamic variables including wall shear stress and velocity exhibited more significant changes [16]. Different finding are reported by Shobayashi et al., where stent deployment induced pressure reductions in aneurysms [17]. While all these results appear contradictory, both instances of change can be theoretically explained by the results of our study. We believe the novelty of this study is that our theory is able to explain consistently both pressure elevation and pressure reduction after stent deployment. Particularly, regarding the relationship between pressure and aneurysm rupture, in addition to various proteases with high proteolytic activity that could participate in degradation of the arterial walls, pressure elevation resulting from flow alternation reportedly acts as a contributing factor in delayed rupture after FD stent deployment [9, 33–41]. In addition, the pressure effect on the aneurysmal surface may be involved, with Suzuki et al. suggesting pressure difference as a parameter to consider regarding the presence of thin regions of the aneurysmal wall [18]. Considering the possibility that pressure elevation represents a risk factor for aneurysmal rupture, it is important that surgeons consider more effective and safe FD stent deployments, decreasing the risk of elevated intra-aneurysmal pressure by selecting stent mesh density based on aneurysm size. Indeed, it has been reported that additional coil deployments can also provide a useful way to avoid delayed rupture [9]. It has to be understood, that the absolute value of the pressure shown in this study is not a meaningful physical value itself since such values will change depending on the boundary condition and location. However, our approach makes it possible to quantitatively compare the relative changes in pressure for the same aneurysm since the geometry and boundary condition are the same.

On the other hand, regarding blood flow stagnation, although *RVR* did not increase significantly until the wire mesh density reached approximately 20% in the Giant aneurysm model, we observed that the pressure increased until the wire mesh density reached 27.5% from Fig 4C and 4D. Such results imply that when treating a large aneurysm with a stent, use of a stent with low wire mesh density or a local decrease in wire mesh density due to deployment in a curved artery may not only provide sufficient blood flow stagnation, but may also increase the risk of elevating aneurysm pressure. Our results thus indicate the need for careful selection of stents when treating larger aneurysms. That implies for surgeons to consider the risk of pressure elevation and eventually deploy coils depending on the situation. It is important to perform stent deployments with the understanding that eventual indiscriminate stenting for such aneurysms may increase the risk of rupture, even if good stent apposition is achieved. CFD analysis may provide an effective tool for proactively controlling the risk of such dangerous hemodynamic alterations during stent deployment. In addition, these CFD results may prove useful in designing stents for specific purposes.

### Limitations

This study obtained clearly indicative results for hemodynamic characteristics in relation to aneurysm size and stent design deployed. However, some limitations regarding CFD analysis and the models of aneurysms and stents generated must be considered. First, the geometry of the basic aneurysm model was designed based on cavernous aneurysms of the internal carotid artery, since intracranial stents including FDs are often deployed in the internal carotid artery. Stent deployment and its effect on bifurcation-type aneurysms was not tested in this study. In addition, we have not explored variables of aneurysm design such as angle of the aneurysm to the parent artery, or aneurysm shape. Although our research findings could not be directly generalized for all types of aneurysms due to the use of a single basic aneurysm model, we believe that our findings will be useful to understand the basic trends of hemodynamic changes depending on the combination of aneurysm size and stent wire mesh density. As a result, this will allow next to expand methodologically the research into the effects of aneurysm geometrical characteristics such as typical and irregular shapes or blebs and ultimately to select the most suitable FD stents for each aneurysm. Further research into a wider variety of aneurysms is clearly needed. Second, as this study applied a Newtonian blood model, average velocity in the aneurysmal dome may decrease more when a non-Newtonian model is applied, especially in cases of Large or Giant aneurysm. Third, the assumption that the arteries represent a rigid body is also a limitation. The geometry of cerebral arteries sometimes deforms due to the force resulting from stent deployment [42]. Such deformation could possibly change the main blood flow completely (i.e., alter aneurysm inflow). The effects of these limitations on the analysis and results needs to be investigated by an in vitro study, such as measurement of particle imaging velocimetry. We also have not accurately estimated the range of possible error by introducing a rigid body. Nevertheless, these methods including the use of a Newtonian blood model and a rigid body have been reported as useful methods for obtaining an initial understanding of the basic hemodynamic characteristics in an aneurysm [43–46]. Moreover, patient-specific research should be performed to elucidate the relationships between hemodynamic changes and complications after stent deployment. In particular, the inflow condition may be altered due to the change of local pressure resistance after stent deployment [47]. Although we need patient-specific inflow data to analyze such situation, since our present study used the basic geometry model, we have applied a unified inflow boundary condition referring to our previous study. We believe that this is a reasonable assumption to obtain the basic trends of hemodynamics [48, 49]. Although our research indicated that aneurysmal pressure would elevate or drop depending on aneurysm size and type of stent deployed, whether complications are triggered from such changes in hemodynamic parameters remains unclear. However, it is important to know that undesirable hemodynamic phenomena, such as pressure elevation may occur depending on the type of deployed stent and the size of the aneurysm. In addition, some hemodynamic parameters such as wall shear stress or oscillatory shear index were not under main consideration in this study. These parameters were used to visualize the change of flow characteristics with subtle differences in the aneurysm or parent artery shape that occurred in the natural history. On the other hand, when a powerful flow diverting device such as a stent is evaluated, we consider that it is more useful to focus on the basic parameters such as velocity or pressure, which are fundamental elements in fluid mechanics. The stent used in this study was a simple model in which stent wires pass through each other, whereas actual stents use wires that alternately intertwine and are braided. However, previous studies have reported that the differences between this simplified model and stricter models appear negligible [50]. In addition, our present methodology to generate the stent could not simulate an actual stent apposition (i.e., although our method allows for ideal stent deployment without any gap between stent and the parent artery, the good stent apposition is not guaranteed also during real deployments). Although we believe that this limitation will not interfere with the understanding of the basic trend, this is also suitable as a the further discussion point when a patient-specific study is conducted. Furthermore, stents in this study were not designed based on commercially available intracranial stents. More specific research considering such stents (e.g., PED, Enterprise, etc.) should be performed in future studies.

## Conclusion

We performed CFD analysis on 5 basic aneurysm models of different sizes and deployed stents with 6 different wire mesh densities for each aneurysm. Although we still have the limitation that only basic cavernous aneurysm models were investigated, observing velocity in the aneurysm, volume flow rate into the aneurysm, pressure on the aneurysmal surface, and residual volume flow in the aneurysm yielded some important background findings:

- Velocity and volume flow rate decreased with increasing wire mesh density of the stent deployed.
- Due to the balance of hemodynamic loss between inflow and outflow, intra-aneurysmal pressure increased then dropped as wire mesh density increased in Medium-Large, Large, and Giant aneurysms, although Small and Medium aneurysms showed continuous decreases in pressure.
- Residual flow volume after stent deployment did not significantly increase when a stent with low wire mesh density was applied to a Giant aneurysm, despite obvious reductions in velocity and volume flow rate.
- Selection of appropriate stents according to aneurysm size may contribute to reduced risks of hemodynamic alternations related to stent deployment.
